# Evaluation of clinical efficacy of Chemotherapy for Rhabdomyosarcoma in children

**DOI:** 10.12669/pjms.36.5.1829

**Published:** 2020

**Authors:** Zhaohui Ning, Xiping Liu, Guang Qin, Lei Wei, Xia Li, Jingjing Shen

**Affiliations:** 1 Zhaohui Ning, Department of Traditional Chinese Medicine, Taian City Central Hospital, Shandong, 271000, China; 2 Xiping Liu, Department of Tumor Minimally Invasive, Taian City Central Hospital, Shandong, 271000, China; 3 Guang Qin, Department of Oncology, Taian City Central Hospital, Shandong, 271000, China; 4 Lei Wei, Department of Traditional Chinese Medicine, Taian City Central Hospital, Shandong, 271000, China; 5 Xia Li, Department of Pediatric, Taian City Central Hospital, Shandong, 271000, China; 6 Jingjing Shen, Department of Pediatric, Taian City Central Hospital, Shandong, 271000, China

**Keywords:** Children, Rhabdomyosarcoma, Clinical features, Chemotherapy, Recurrence, Efficacy

## Abstract

**Objective:**

To investigate the clinical characteristics and treatment methods of rhabdomyosarcoma in children and the efficacy of the methods.

**Methods:**

The clinical data of 30 children with rhabdomyosarcoma who were admitted to our hospital from August 2013 to August 2017 were retrospectively analyzed. The clinical characteristics were summarized, and the curative effect and prognosis were evaluated.

**Results:**

Among all the children (N=30), there were 20 males and 10 females, with a median age of 3.5 years. As to the primary site, there were 13 cases of head and neck, 11 cases of trunk, three cases of urogenital system and three cases of limbs. There were 25 cases of embryonic type, 4 cases of alveolar type and one case of polymorphic type. As regards clinical stage, there were one case of stage I, 9 cases of stage II, 13 cases of stage III and 7 cases of stage IV. There were one case of low risk, 19 cases of medium risk and 10 cases of high risk. Eight cases received surgery alone, 22 cases received combined treatment of surgery and chemotherapy (the chemotherapeutics followed three schemes, low-risk group (VAC+VA), moderate risk group (VAC) and high risk group (alternating use of VDC and IE). Among all the cases (N=30), there were 14 cases of complete remission (CR), five cases of partial remission (PR), four cases of stable disease (SD), and 7 cases of progressive disease (PD). The CR rate was (N=14, 46.7%). The three-year overall survival (OS) rate was (N=19, 63.3%). The clinical efficacy and prognosis of children receiving surgery and chemotherapy were better than those of children receiving surgery alone, and the difference was statistically significant (P<0.05).

**Conclusion:**

Rhabdomyosarcoma in children frequently happens in the head, neck and trunk. Embryonic type is the main pathological type of rhabdomyosarcoma. Comprehensive and standardized treatment based on surgery and chemotherapy is an important way to improve the curative effect in the treatment of rhabdomyosarcoma in children.

## INTRODUCTION

Rhabdomyosarcoma (RMS) is a kind of soft tissue malignant tumors originating from undifferentiated primary mesenchymal cells with the potential to differentiate into striated muscle cells, which has an incidence of 4.5% among all pediatric tumors. RMS is the third largest child malignant tumors after neuroblastoma and nephroblastoma and also the most common type of soft tissue sarcoma in children, with an incidence of 50%. It can occur in any part of the body, and its clinical manifestations are diverse.^1^-^3^

It is generally believed that RMS is derived from muscle stem cells or pluripotent mesenchymal stem cells.^4^ RMS is composed of rhabdomyoblasts with different degrees of differentiation, which belongs to small blue round cell tumor. Immunohistochemistry plays an important role in the diagnosis of RMS. Specific proteins related to skeletal muscle are helpful to distinguish other tumors, including actin, myosin, desmin, Myglobin, Z-band protein, MyoD and mygenin.^5^ The pathological types of RMS in children are mainly embryonal rhabdomyosarcoma (ERMS) and alveolar rhabdomyosarcoma (ARMS); grape-shaped type and spindle cell type are variant of ERMS, and polymorphous cell type is rare. The incidence of ERMS among children with RMS was 70%, which is characterized by tumor cells similar to immature skeletal muscle cells under light microscope. RMS grows rapidly, has a high degree of malignancy, and is sensitive to chemotherapy and radiotherapy, but the effect of single treatment is poor; therefore, comprehensive treatment such as surgery, chemotherapy and radiotherapy are needed.^6^,^7^

The United States has established Intergroup RMS Study Group (IRSG) in 1972 to study RMS among teenagers under the age of 20. It has carried out clinical studies of IRS stage I-IV and formed a regular diagnostic and treatment system. In the IRS-IV study, the three-year failure free survival (FFS) and overall survival (OS) of RMS were 77% and 86% respectively.^8^ In the study which was carried out by the International Society of Paediatric Oncology (SIOP) in European countries, the five-year FFS and OS of RMS were 60% and 74% respectively.^9^ Compared to the United States and European, the RMS diagnostic and treatment in China has defects.^10^ In this study, the clinical data of 30 children with RMS who were received and treated between August 2013 and February 2016 were retrospectively analyzed. The clinical characteristics, treatment methods, curative effects and prognosis of these children were summarized. This work provides a clinical basis for the standardization of RMS diagnosis and treatment and the improvement of curative effect of RMS.

## METHODS

This is a retrospective study. The sample size was calculated using the following formula:


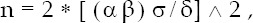


where δ is the required distinction degree, σ stands for the overall standard deviation or its estimated value s, α and β can be looked up from the row of free degree υ = ∞ − in the table of t critical value.

The clinical data of 30 children with RMS who were admitted to Taian City Central Hospital from August 2013 to February 2016 were collected. Inclusion criteria was those receiving comprehensive treatment such as surgery or surgery combined with chemotherapy, having definite pathological diagnosis after surgery, having complete clinical data and follow-up records, having the cause of death relating to the tumor, and having informed consent signed by the guardian of the child. Exclusion criteria was having heart, liver and kidney dysfunction, having coagulation disorders, and having chemotherapy contraindications. This study was approved by the Medical Ethics Committee of our hospital on August 29, 2019.

### Clinical diagnosis and staging

All cases were pathologically confirmed by tumor biopsy or tumor resection. Thirty children were examined by contrast-enhanced computed tomography (CT) or Magnetic Resonance Imaging (MRI), chest CT, cranial MRI, bone scan and bone marrow examination. RMS is divided into stage I-IV according to the staging standards of IRS. Considering the histopathological subtypes, RMS was divided into low risk group^11^ (stage I non-alveolar RMS), moderate risk group (stage I, II and III alveolar RMS, stage II and III non-alveolar RMS) and high risk group (stage IV alveolar RMS and non-alveolar RMS).

### Treatment plan

Surgical treatment was selected for those whose tumors were evaluated as being capable of completely resected by surgical evaluation. Those who had resection difficulties or needed to retain organ function were given adjuvant chemotherapy after biopsy diagnosis. RMS 2002 scheme of Shanghai Children’s Medical Center was combined with the scheme of IRS, and the details are as follows.

### Low-risk group (VAC+VA)

VAC scheme: vincristine was used at a dose of 1.5 mg/m^2^ at Day 1, 8, 15; cyclophosphamide was used at a dose of 1.2 g/m^2^ at Day 1; actinomycin D was used at a dose of 0.045 mg/kg at Day 1; chemotherapy was performed at the 1^st^, 7^th^, 13^th^, 19^th^, 25^th^ and 31^st^ week. VA scheme: vincristine was used at a dose of 1.5 mg/m^2^ at Day 1, 8 and 15; actinomycin D was used at a dose of 0.045 mg/kg at the first day; chemotherapy was performed at the 4^th^, 10^th^, 16^th^, 22^nd^, 28^th^ and 34^th^ week.

### Middle-risk group (VAC)

VAC scheme: vincristine was used at a dose of 1.5 mg/m^2^ at Day 1, 8 and 15; cyclophosphamide was used at a dose of 1.2 g/m^2^ at the first day, actinomycin D was used at the dose of 0.045 mg/kg at the first day. The treatment was repeated every three weeks, for 44 weeks.

### High-risk group (alternation of VDC and IE)

VDC scheme: vincristine was used at the dose of 1.5 mg/m^2^ at Day 1, 8, 15; adriamycin was used at the dose of 30 mg/m^2^ at Day 1 and 2; cyclophosphamide was used at the dose of 1.2 g/m^2^ at Day 1. IE scheme: isocyclophosphamide was used at the dose of 1.8 g/m^2^ at Day 1-5; etoposide was used at the dose of 100 mg/m^2^ at Day 1-5. The two schemes alternated for 48 weeks.

During the treatment, all the children were given clinical pathway nursing. Specific methods are as follows. According to the different conditions of the children, the detailed clinical pathway nursing scheme was formulated, and nursing was carried out strictly in accordance with the nursing pathway table. The first step was admission nursing. On the day of admission, the children and their families were introduced with the hospital environment, mainly including medical environment, hospitalization environment, nurses, matters needing attention, in order to alleviate the children’s negative psychology such as tension and fear and to establish a good nurse-patient relationship. The second step was preoperative nursing. Psychological counseling for children and their parents could help children quickly adapt to the hospitalization environment, eliminate their negative psychology and improve their treatment compliance. Parents of children who had undergone the operation were invited to explain their experience, so as to alleviate the preoperative tension of the children and their families and promote the smooth operation. The third step was postoperative nursing. After operation, the changes of heart rate and blood pressure were closely observed and explained to the parents that vomiting and nausea were likely to occur before awake. After the anesthesia effect disappeared, there would be irritability, crying and other phenomena. Pain factors were carefully examined, children’s attention was distracted by telling stories and singing, so as to alleviate their pain. The parents were informed with dietary precautions and guided to supplement nutrition for children.

### Therapeutic effect analysis and follow-up

The therapeutic effect was evaluated according to Response Evaluation Criteria in Solid Tumors (RECIST),^12^ including complete remission (CR), partial remission (PR), stable disease (SD) and progressive disease (PD).

Re-examination or follow-up by telephone was carried out. Three cases were followed up by telephone, and the remaining 27 cases were followed up by outpatient or inpatient follow-up. The follow-up period was from the time of first hospitalization to February 2018. The follow up continued until there was recurrence, death or abandon.

### Statistical analysis

SPSS 24.0 was used for statistical analysis. Kaplan Meier method was used to calculate the survival rate. Log-Rank test was used to compare the survival rate between groups. Chi-square test was used in processing counting data, and t-test was used for measuring data. P<0.05 indicated that the difference was statistically significant.

## RESULTS

### Clinical characteristics

**Fig.1:**
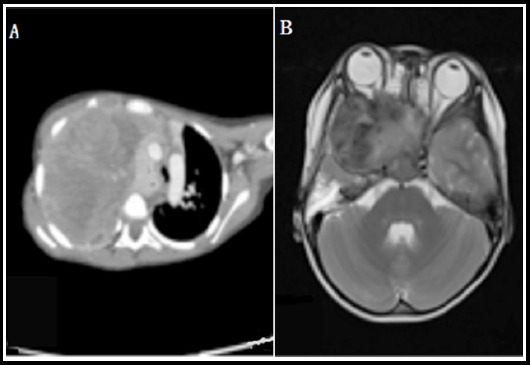
Imaging data of children with RMS at different sites. **A:** Thoracic CT, **B:** Cranial MRI.

Among all the children (N=30), there were 20 males and 10 females, with a median age of 3.5 years (3 months~8 years). As to the position of the tumors, there were 13 cases of head and neck, 11 cases of trunk, three cases of urogenital system and three cases of limbs. Some imaging data are shown in Fig.1. As to initial symptoms, the incidence of ocular symptoms (exophthalmos, decreased vision, lacrimation, etc.) was 20% (6/30), the incidence of nasal symptoms (nasal obstruction, epistaxis, etc.) was 10% (3/30), the incidence of urinary retention was 6.7% (2/30), the incidence of local mass was 60% (18/30), and the incidence of other symptoms (hearing loss, vomiting) was 3.3% (1/30). Among them, there were 25 cases of embryonic type, four cases of alveolar type and one case of polymorphic type. There was no evidence of bone marrow metastasis in all 30 cases. Bone scan result was positive in three cases; the metastatic site was iliac bone, right lower tibia and skull respectively. There were two cases of lung metastasis, two cases of peritoneal implantation, and one case of distant lymph node metastasis. As to the IRS clinical stage, there were one case of stage I, 9 cases of stage II, 13 cases of stage III, and seven cases of stage IV. There were one case of low risk, 19 cases of medium risk and 10 cases of high risk. Eight cases received surgery alone, and 22 cases received combined treatment of surgery and chemotherapy.

### Clinical efficacy

Among the children (N=30), the CR rate of the group of surgery combined with chemotherapy was 54% (12/22), and the three-year OS rate was 72.7% (16/22); the CR rate of the surgery group was 25.0% (2/8), and the three-year OS rate was 37.5% (3/8). Compared with the surgery group, the CR rate and OS rate of the group of surgery combined with chemotherapy were significantly higher (P<0.05).

### Survival function analysis

**Fig.2:**
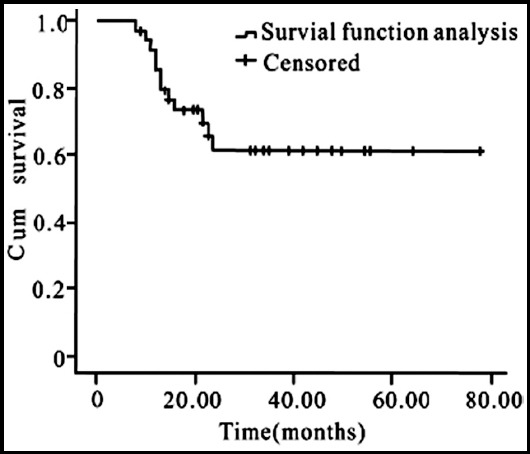
Survival function analysis of 30 cases

**Fig.3:**
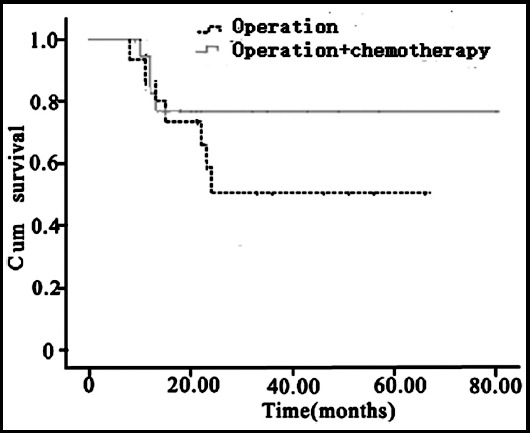
Survival function analysis of different treatment methods.

The follow-up stopped at February 2019. The median follow-up period was 39 months (8~80 months). At the end of follow-up, five out of the eight children who underwent surgery had recurrence, of which one patient received chemotherapy after recurrence but still progressed; 10 out of the 22 patients who underwent surgery and chemotherapy had recurrence. There were 14 cases of CR, 5 cases of PR, four cases of SD, and seven cases of PD. The CR rate was (N=14, 46.7%), and the 3-year OS rate was (N=19, 63.3%). Statistical analysis of survival function showed that the total survival time of the 30 children was (53.63±5.75) months, and the 3-year OS rate was (N=19, 63.3%), as shown in Fig.2. Survival function analysis of different treatment methods is shown in Fig.3.

## DISCUSSION

RMS is the most common soft tissue malignant tumor in childhood, ranking the third among malignant solid tumors in children. Most of them occur in head and neck, trunk, limbs and urogenital system. The main pathological type of RMS is embryonic type.^13^ RMS lacks specific clinical manifestations and is often manifested as exophthalmos, painless mass and urinary retention. Its malignant degree is high, its growth is rapid, and it shows infiltration growth and early metastasis.^14^ In this study, 13 cases had tumors in head and neck and 11 had tumors in the trunk, accounting for 80.6%, which were the high-incidence sites. Among them, 25 cases were embryonic type, accounting for 83.3%, which was the main pathological type, and it was consistent with the results reported in previous study.^15^

In recent years, with the application of multidisciplinary comprehensive treatment, the overall long-term OS rate of children with RMS has been greatly improved. At present, the long-term OS rate of RMS children in the world has reached more than 70%.^16^ However, according to reports in China, the 5-year OS rate of RMS patients in China is 40%~65%, and the recurrence rate is high, which has a gap compared with foreign countries.^17^ In this study, the median follow-up time was 39 months, the OS rate was 63.3%, and the 5-year estimated survival rate was 46.5%, which was lower than that of foreign reports. The reason might be related to the high incidence of distant metastasis, the single treatment method and the irregular management.

RMS has high malignancy and poor curative effect. It needs comprehensive treatment such as surgery and chemotherapy.^18^ Surgery can be used only when the whole tumor can be resected or there is a little residue. If tumors cannot be completely resected or organs and their functions need to be preserved, chemotherapy can be used to reduce the size of the tumor before surgery. The role of adjuvant chemotherapy cannot be ignored. Preoperative chemotherapy can effectively reduce the volume of tumors and remove potential metastatic lesions, thus providing a good basis for radical surgery.^19^ Liu et al. analyzed the effect of preoperative chemotherapy on 12 cases of bladder RMS and found that preoperative chemotherapy could significantly reduce the volume of bladder RMS,^20^ which laid a foundation for preserving bladder function and complete resection of bladder tumors. Postoperative chemotherapy plays an important role in preventing recurrence of tumors. In this study, the CR and OS rates in the group of surgery combined with chemotherapy were higher than those in the group which did not undergo chemotherapy, which confirmed that adjuvant chemotherapy played an important role in the control of RMS.

It is well known that standardized and effective chemotherapy is helpful to remove the residual lesions of cancer patients after operation, and children are more sensitive and tolerant to chemotherapeutic drugs than adults. Thus the recurrence rate can be reduced by chemotherapy with regular cycle and effective scheme in clinic.^21^ The results of this study showed that 22 children received surgery and chemotherapy, of which 10 cases had recurrence (45.5%). Compared with children who did not undergo chemotherapy (5/8, 62.5%), the recurrence rate of the group of surgery combined with chemotherapy was significantly lower, which confirmed that chemotherapy played an important role in the prevention and treatment of children’s RMS recurrence.

In addition, in this study, compared with the surgery group, the CR rate and 3-year OS rate in the group of surgery combined with chemotherapy were significantly higher, and the difference was statistically significant. Therefore, comprehensive and standardized treatment plan is an important way to improve the diagnosis and treatment effect of RMS.

## CONCLUSION

At present, the overall prognosis of children with RMS in China has been significantly improved, but the recurrence rate is still high. The prognosis of patients with recurrent RMS is poor. The survival rate after recurrence depends on the clinical and pathological characteristics at the initial diagnosis and the first treatment method. Applying local treatment measures such as individualized chemotherapy, radical surgery and/or radiotherapy according to specific conditions can reduce the recurrence rate and improve the prognosis of patients with RMS.

### Authors’ Contribution

**ZHN, XPL & JJS:** Study design, data collection and analysis.

**XPL, GQ, LW & XL:** Manuscript preparation, drafting and revising.

**ZHN & JJS:** Review and final approval of manuscript, are also responsible for integrity of the study.

## References

[1] Hickman PE, Messina S, Trotter JM, Masarei JR (1983). High creatine kinase MB isoenzyme activity associated with a rhabdomyosarcoma. Clin Chem.

[2] Jr LW, Hays DM, Heyn R, Tefft M, Crist W, Beltangady M (2015). Lymphatic metastases with childhood rhabdomyosarcoma. A report from the Intergroup Rhabdomyosarcoma Study. Cancer.

[3] Affinita MC, Ferrari A, Milano GM, Scarzello G, De Leonardis F, Coccoli L (2018). Long-term results in children with head and neck rhabdomyosarcoma:A report from the Italian Soft Tissue Sarcoma Committee. Pediatr Blood Cancer.

[4] Arnold MA, Barr FG (2017). Molecular diagnostics in the management of rhabdomyosarcoma. Expert Rev Mol Diagn.

[5] Mizumoto M, Murayama S, Akimoto T, Demizu Y, Fukushima T, Ishida Y (2018). Preliminary results of proton radiotherapy for pediatric rhabdomyosarcoma:a multi-institutional study in Japan. Cancer Med.

[6] Liu YT, Wang CW, Hong RL, Kuo SH (2019). Prognostic factors and treatment outcomes of adult patients with rhabdomyosarcoma after multimodality treatment. Anticancer Res.

[7] Agaram NP, Zhang L, Sung YS, Cavalcanti M, Torrence D, Wexler L (2019). Expanding the spectrum of intraosseous rhabdomyosarcoma:correlation between 2 distinct gene fusions and phenotype. Am J Surg Pathol.

[8] Sangkhathat S (2015). Current management of pediatric soft tissue sarcomas. World J Clin Pediatr.

[9] Vaarwerk B, van der Lee JH, Breunis WB, Orbach D, Chisholm JC, Cozic N (2018). Prognostic relevance of early radiologic response to induction chemotherapy in pediatric rhabdomyosarcoma:A report from the International Society of Pediatric Oncology Malignant Mesenchymal Tumor 95 study. Cancer.

[10] Miao Z, Wang XJ, Sun J, Zhu YN, Wang G, Zou X (2017). Analysis on diagnosis and treatment of 17 cases of children rhabdomyosarcoma. J China Pediatr Blood Cancer.

[11] Vickie YJ, Christopher DM (2014). WHO classification of soft tissue tumors:an update based on the 2013 (4th) edition. Pathology.

[12] Pediatric Oncology Professional Committee of Chinese Association against Cancer. Recommendations for diagnosis and treatment of rhabdomyosarcoma in Children and adolescents in China (CCCG-RMS-2016) (2017). Chin J Pediatr.

[13] Saltzman AF, Cost NG (2018). Current treatment of pediatric bladder and prostate rhabdomyosarcoma. Curr Urol Rep.

[14] Casey DL, Wolden SL (2018). Rhabdomyosarcoma of the head and Neck:Amultimodal approach. J Neurol Surg B Skull Base.

[15] Ma X, Huang D, Zhao W, Sun L, Xiong H, Zhang Y (2015). Clinical characteristics and prognosis of childhood rhabdomyosarcoma:A ten-year retrospective multicenter study. Int J Clin Exp Med.

[16] Li Y, Bakke J, Finkelstein D, Zeng H, Wu J, Chen TS (2018). HNRNPH1 is required for rhabdomyosarcoma cell growth and survival. Oncogenesis.

[17] Tang X, Guo X, Yang X, Wan Z, Ai Y, Jiang MY (2016). Clinical features and therapeutic efficacy of childhood rhabdomyosarcoma. Chin J Obstetr Gynecol Pediatr.

[18] Bayramoglu Z, Kebudi R, Yilmaz R, Bay SB, Kebudi A, Karanlik H (2018). Primary rhabdomyosarcoma of the breast:imaging findings and literature review. Breast Care.

[19] Jarząb A, Łuszczki J, Guz M, Skalicka-woźniak K, Hałasa M, Smok-kalwat J (2018). Combination of osthole and cisplatin against rhabdomyosarcoma TE671 cells yielded additive pharmacologic interaction by means of isobolographic analysis. Anticancer Res.

[20] Liu JH, Lin T, He DW, Liu F, Hua Y, Liu X (2015). Effects of preoperative chemotherapy in children with bladder rhabdomyosarcoma. Chin J Pediatr Surg.

[21] Misir AF, Zerener T, Günhan O (2014). Dental management long term follow-up of the post radio-chemotherapy-Rhabdomyosarcoma patient:Report of a case. J Oral Maxillofac Surg Med Pathol.

